# Three doses of a recombinant conjugated SARS-CoV-2 vaccine early after allogeneic hematopoietic stem cell transplantation: predicting indicators of a high serologic response—a prospective, single-arm study

**DOI:** 10.3389/fimmu.2023.1169666

**Published:** 2023-04-19

**Authors:** Maryam Barkhordar, Bahram Chahardouli, Alireza Biglari, Mohammad Ahmadvand, Tanaz Bahri, Farshid Alaeddini, Leyla Sharifi Aliabadi, Seied Saeid Noorani, Fahimeh Bagheri Amiri, Mohammad Biglari, Mohammad Reza Shemshadi, Ardeshir Ghavamzadeh, Mohammad Vaezi

**Affiliations:** ^1^ Cell Therapy and Hematopoietic Stem Cell Transplantation Research Center, Research Institute for Oncology, Hematology, and Cell Therapy, Tehran University of Medical Sciences, Tehran, Iran; ^2^ Department of Medical Genetics, Tehran University of Medical Sciences, Tehran, Iran; ^3^ Tehran Heart Center, Tehran University of Medical Sciences, Tehran, Iran; ^4^ Department of Epidemiology and Biostatistics, Research Centre for Emerging and Reemerging Infectious Diseases, Pasteur Institute of Iran, Tehran, Iran; ^5^ Cancer & Cell Therapy Research Center, Tehran University of Medical Sciences, Tehran, Iran

**Keywords:** hematopoietic stem cell transplantation, SARS-CoV-2, RBD subunit vaccine, conjugate vaccine, humoral response, T-cell response, immunogenicity predictors

## Abstract

**Background:**

Allogeneic hematopoietic stem cell transplant (allo-HSCT) recipients must be vaccinated against SARS-CoV-2 as quickly as possible after transplantation. The difficulty in obtaining recommended SARS-CoV-2 vaccines for allo-HSCT recipients motivated us to utilize an accessible and affordable SARS-CoV-2 vaccine with a recombinant receptor-binding domain (RBD)–tetanus toxoid (TT)-conjugated platform shortly after allo-HSCT in the developing country of Iran.

**Methods:**

This prospective, single-arm study aimed to investigate immunogenicity and its predictors following a three-dose SARS-CoV-2 RBD–TT-conjugated vaccine regimen administered at 4-week (± 1-week) intervals in patients within 3–12 months post allo-HSCT. An immune status ratio (ISR) was measured at baseline and 4 weeks (± 1 week) after each vaccine dose using a semiquantitative immunoassay. Using the median ISR as a cut-off point for immune response intensity, we performed a logistic regression analysis to determine the predictive impact of several baseline factors on the intensity of the serologic response following the third vaccination dose.

**Results:**

Thirty-six allo-HSCT recipients, with a mean age of 42.42 years and a median time of 133 days between hematopoietic stem cell transplant (allo-HSCT) and the start of vaccination, were analyzed. Our findings, using the generalized estimating equation (GEE) model, indicated that, compared with the baseline ISR of 1.55 [95% confidence interval (CI) 0.94 to 2.17], the ISR increased significantly during the three-dose SARS-CoV-2 vaccination regimen. The ISR reached 2.32 (95% CI 1.84 to 2.79; *p* = 0.010) after the second dose and 3.87 (95% CI 3.25 to 4.48; *p* = 0.001) after the third dose of vaccine, reflecting 69.44% and 91.66% seropositivity, respectively. In a multivariate logistic regression analysis, the female sex of the donor [odds ratio (OR) 8.67; *p* = 0.028] and a higher level donor ISR at allo-HSCT (OR 3.56; *p* = 0.050) were the two positive predictors of strong immune response following the third vaccine dose. No serious adverse events (i.e., grades 3 and 4) were observed following the vaccination regimen.

**Conclusions:**

We concluded that early vaccination of allo-HSCT recipients with a three-dose RBD–TT-conjugated SARS-CoV-2 vaccine is safe and could improve the early post-allo-HSCT immune response. We further believe that the pre-allo-HSCT SARS-CoV-2 immunization of donors may enhance post-allo-HSCT seroconversion in allo-HSCT recipients who receive the entire course of the SARS-CoV-2 vaccine during the first year after allo-HSCT.

## 1 Background

The novel coronavirus disease 2019 (COVID-19), caused by the severe acute respiratory syndrome coronavirus (SARS-CoV-2), has generated a severe medical crisis. Immunodeficiency after allogeneic hematopoietic stem cell transplant (allo-HSCT) increases the susceptibility of the recipient to the most severe SARS-CoV-2 infection and a greater fatality rate than the general population (1, 2). The timely vaccination of hematopoietic stem cell transplant (allo-HSCT) patients can boost immunity, decreasing the morbidity and mortality associated with COVID-19.

Although immune responses to vaccination are frequently restricted and uncertain in the initial phases of allo-HSCT (3, 4), some professional bodies, notably the European Society for Blood and Marrow Transplantation (EBMT), advocate prophylactic vaccination as quickly as 3 months after the transplant to provide initial immune protection (5, 6). The most widely used vaccination platforms in allo-HSCT recipients were mRNA vaccines, such as BNT162b2 from Pfizer-BioNTech and mRNA-1273 from Moderna, and adenoviral vector vaccines, such as Ad26.COV2.S from Johnson & Johnson and ChAdOx1-S from AstraZeneca (7, 8). In Iran, we followed the EBMT recommendation for post-allo-HSCT SARS-CoV-2 vaccination; however, owing to limited access to mRNA-based platforms, we mainly utilized available vaccines, including inactivated platforms (e.g., the Sinopharm vaccine), for allo-HSCT recipients.

Recent investigations, however, have demonstrated that many allo-HSCT patients, particularly those vaccinated soon after allo-HSCT, reacted poorly to two doses of the mRNA vaccine (7, 8). The serologic response was 32% [95% confidence interval (CI) 15%–50%] for patients vaccinated 6 months post transplantation (9, 10), 50% (95% CI 42% to 61%) for patients vaccinated between 6 and 12 months post transplantation (11–13), and 87.9% (95% CI 72% to 95%) for patients vaccinated after 1 year following allo-HSCT (12–14). However, further research showed that giving the third dose of the SARS-CoV-2 vaccine markedly improved the serological response after allo-HSCT (15–18).

The protein subunit platform, based on SARS-CoV-2 protein components, such as the spike protein (S1) and receptor-binding domain (RBD), is a different vaccination technology that has demonstrated advantages in terms of tolerability, efficacy, and cost (19). According to published data, RBD-based SARS-CoV-2 vaccines, such as Abdala, Zhifei, and Noora, have shown promising results in healthy people (20–22). Furthermore, as demonstrated in preclinical investigations, humoral and cellular immune responses were strengthened by coupling RBD with the tetanus toxoid (TT) (23).

Soberana 2, also called PastoCovac, is the first SARS-CoV-2 vaccination using RBD conjugated to TT, manufactured in collaboration between the Cuban Finlay Institute and the Iranian Pasteur Institute. Soberana 2 (PastoCovac) has been certified for emergency use in adults and children aged more than 2 years in Cuba and Iran. This platform is simple to construct and offers benefits in terms of storage and transportation. The safety and immunogenicity of Soberana 2 have previously been studied in dedicated phase 1, 2, and 3 clinical studies (24–26). In a recently published study with autologous HSCT patients, we demonstrated that two doses of the novel RBD–TT-conjugated SARS-CoV-2 vaccine (PastoCovac) given soon after autologous transplants were safe and significantly enhanced the serologic response to a level comparable to the mRNA-based platform, although less than that of the healthy controls (27).

The difficulty in obtaining recommended SARS-CoV-2 vaccines for allo-HSCT recipients, such as the mRNA- or adenoviral vector-based platforms, as well as the necessity for timely immunization of allo-HSCT recipients, prompted us to explore the use of an accessible and affordable (RBD–TT-conjugated) SARS-CoV-2 vaccine early after allo-HSCT. We also examined how the characteristics of the patients and donors and their immunological status against SARS-CoV-2 at the time of allo-HSCT influenced subsequent serologic responses to early post-allo-HSCT vaccination.

## 2 Methods

### 2.1 Study design, registry, and ethical approval

This prospective and single-group clinical trial assessed the immunogenicity and safety of three RBD–TT conjugated SARS-CoV-2 vaccine doses in adult acute leukemia patients who underwent allo-HSCT at the Hematology, Oncology and Stem Cell Transplantation Research Center (HORCSCT) of Tehran University, Tehran, Iran. The study was registered on ClinicalTrial.gov (as NCT05185817) and the Iranian Registry of Clinical Trials (as IRCT20140818018842N22). Recruitment for the trial began in January 2022.

The trial was conducted under the Helsinki Declaration and Good Clinical Practice and was certified by the Ethics Committee of Tehran University’s Hematology, Oncology and Stem Cell Transplantation Research Center (IR.TUMS.HORCSCT.REC.1400.021). Each recipient provided written informed consent for the PastoCovac vaccine (Pasteur Institute, Tehran, Iran) to be administered, blood samples to be collected, and results to be published.

### 2.2 Inclusion criteria

The research included all adult patients with acute myeloid leukemia (AML) or acute lymphoid leukemia (ALL) who had received allo-HSCT within the previous 3–12 months, were older than 18 years, had achieved complete engraftment, and had no documented history of SARS-CoV-2 infection after allo-HSCT.

### 2.3 Exclusion criteria

Having grade 3 or 4 acute graft-versus-host disease (GvHD) or severe extensive chronic GvHD, taking more than 0.5 mg/kg of prednisolone per day, suffering from severe thrombocytopenia or a coagulation disorder, having a history of an allergic reaction to the vaccine’s active ingredients, being unable to provide consent forms, continuing post-allo-HSCT infection, graft rejection, or experiencing a relapse of the underlying disease were all among the exclusion criteria.

### 2.4 Procedures and data collection

**Figure 1:**
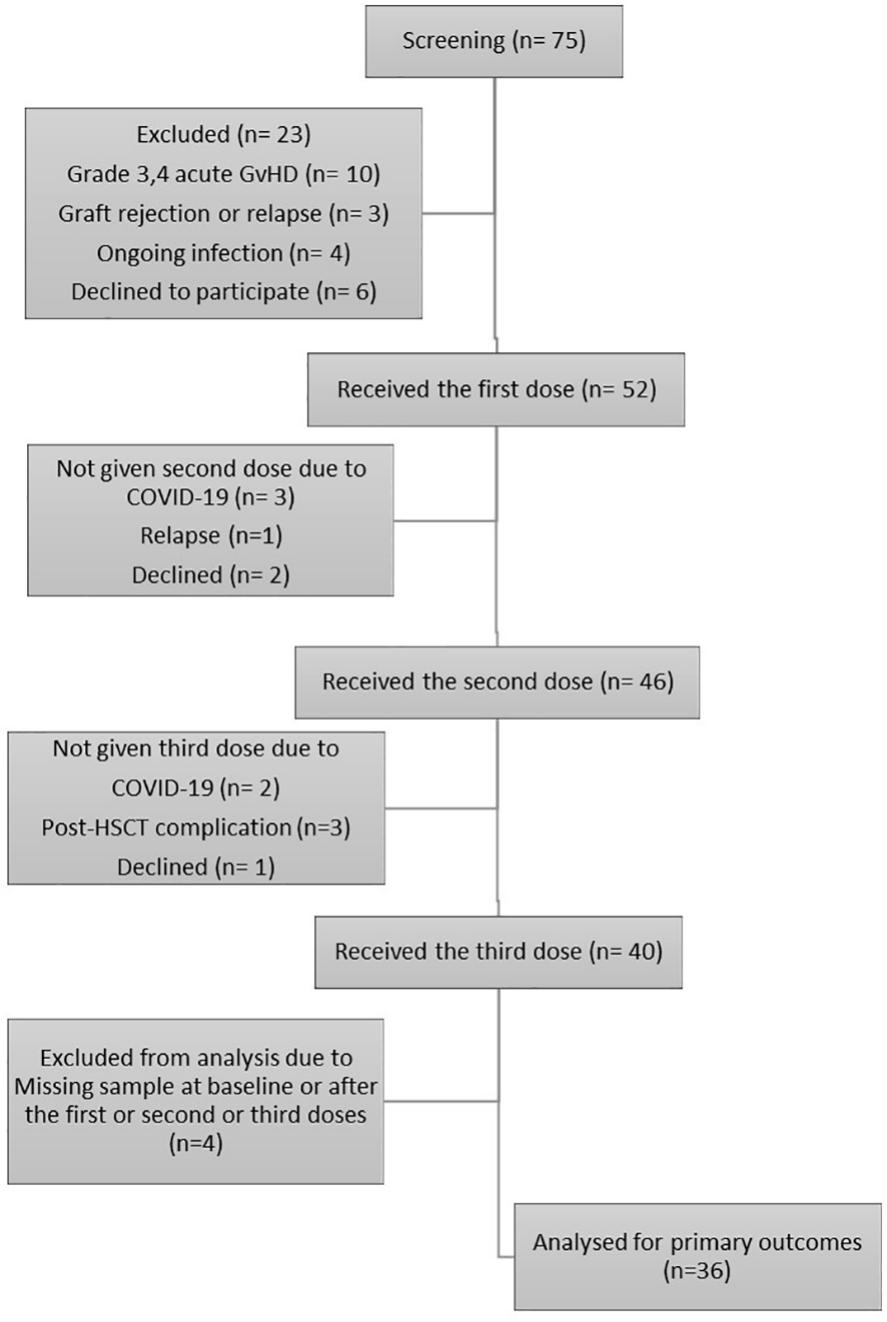
Flowchart of the single-arm study. The chart depicts the subjects screened before the study, those recruited for vaccination, and the processes for selecting or excluding patients.

The research selection flowchart is provided in **Figure 1**. Starting in January 2022, 75 recipients of allo-HSCT were enrolled. A total of 52 people satisfied the eligibility criteria and stayed in the study for post-allo-HSCT vaccination. Acute GvHD, COVID-19 infection, and refusal to volunteer were responsible for most study exclusions. The study ultimately comprised 36 patients who received the three-dose RBD–TT conjugated SARS-CoV-2 vaccine and were given the available serologic tests at four time points: at baseline and after the first, second, and third doses.

Medical personnel administered the vaccination, comprising 0.5 mL of vaccine injected intramuscularly into the deltoid area. We developed an electronic case report form (CRF) using our institution’s web-based software to collect research information, including patients’ and donors’ characteristics, concurrent medications, lymphocyte subpopulation count, and SARS-CoV-2 anti-S1 titers. To evaluate the safety profiles of a new RBD–TT-conjugated SARS-CoV-2 vaccine, we employed active surveillance systems to report any vaccination-related adverse events through daily telenursing calls, which have a higher accuracy than passive monitoring in specific population subgroups (28, 29).

### 2.5 Outcome

The main objectives of our study included the following outcomes:

1. The anti-SARS-CoV-2 spike protein (anti-S) serologic response at 4 weeks (± 7 days) after the third dose of vaccine, defined as an increase in the immune status ratio (ISR) above the cut-off point for a positive result in the semiquantitative test.
2. The predicting factors of a strong immune response following the third vaccination dose, using the median level of ISR as a cut-off point (30).
3. The vaccination’s safety and tolerability up to 1 week after each dosage.

### 2.6 Anti-SARS-CoV-2 antibody evaluation

We used the ChemoBind SARS-CoV-2 Neutralizing Antibody Test Kit (ChemoBind, Tehran, Iran) to measure total antibodies against the receptor-binding domain (RBD) spike protein of SARS-CoV-2 using a semiquantitative immunoassay. Based on the instructions from the manufacturer, an immunoglobulin G (IgG) immune status ratio (ISR) of less than 0.8 is negative, and an IgG ISR greater than 1.1 is positive; ratios between these values are ambiguous and need to be repeated.

All allo-HSCT recipients had their anti-S antibody levels (as ISR) assessed before vaccination and 4 weeks (± 1 week) after receiving the first, second, and third doses of the vaccine. We also measured the pre-allo-HSCT ISR for patients and donors to evaluate the potential predictive impact of the pre-allo-HSCT immune status of patients and donors against SARS-CoV-2 on post-allo-HSCT vaccine-induced antibody production.

### 2.7 Flow cytometry

Peripheral blood samples were collected for all recipients to assess the absolute count and percentage of specific lymphocyte subpopulations at the first (i.e., baseline) and third vaccination doses. The immunophenotype of natural killer (NK), T, and B cells was determined by a 10-color multiparameter flow cytometric analysis of blood samples. The blood samples were collected into ethylenediaminetetraacetic acid (EDTA) tubes and incubated with the following recombinant monoclonal antibodies: anti-CD16 (REA423), anti-CD56 (REA196), anti-CD3 (REA613), anti-CD4 (REA623), anti-CD8 (REA734), anti-CD19 (REA675), and anti-CD45 (REA747). Based on antigen density and brightness, one seven-color panel was designed in pairing markers and fluorochromes: CD16-FITC, CD56-PE, CD3-VioBlue, CD4-PerCP-Vio700, CD8-PEVio770, CD19-APC, and CD45-VioGreen. The experimental controls were unstained, stained with one dye, and fluorescence minus one control.

### 2.8 Safety assessments

Using active surveillance, we reported any reactogenicity adverse effects (AEs), including specific local (pain and swelling at the injection site) or systemic (fever, lethargy, headache, diarrhea, vomiting, and muscle pain) AEs were reported *via* daily telenursing calls for up to 7 days following each vaccination dose. All reactogenicity events were classified as none/mild (grades 0 or 1), moderate (grade 2), severe (grade 3), or life-threatening/death (grades 4 or 5) using the Common Terminology Criteria for Adverse Events (CTCAE) (31). Across the follow-up period, all immunized patients were monitored weekly through phone calls or clinical appointments to identify any occurrences of new or worsening GVHD, a diagnosis of COVID-19, a relapse of underlying disease, or cytopenia until 20 December 2022.

### 2.9 Statistical analysis

The generalized estimating equation (GEE) model was used for assessing the dynamics of the serologic response following each vaccine dose overall and based on the main variables. The predictive impact of confounding factors on the GEE model was then determined by univariate and multivariable analysis.

Using the median ISR as a cut-off point for immune response intensity, we performed a logistic regression analysis to determine the predictive impact of several baseline factors on the intensity of the serologic response following the third vaccination dose. Factors correlated with a vigorous immunological response in the univariate analysis (*p* ≤ 0.20) were then entered into the multivariable model with stepwise forward selection.

The Shapiro–Wilk test was used for assessing the normal distribution of quantitative variables. All tests were two-way, and a *p*-value of less than 0.05 was considered statistically significant. GraphPad Prism version 8 was used to create the graphs (GraphPad Software Inc., San Diego, CA, USA). All statistical analyses were performed using IBM SPSS Statistics, version 23.0 (IBM Corporation, Armonk, NY, USA).

## 3 Results

### 3.1 Patient characteristics

The study included 36 allo-HSCT individuals who received three PastoCovac doses and four serologic tests of blood samples with which to assess the trial’s main end points (**Figure 1**). The study comprised 15 females (41.7%) and 21 males (58.3%), with a mean age of 42.42 years (SD 15.84 years), as shown in **Table 1**. Regarding participants’ primary diseases, 27 patients with AML (75%) and nine with ALL (25%) were included in the trial. All recipients were given the same myeloablative conditioning regimen of busulfan and cyclophosphamide (Bu/Cy) and the same graft source of peripheral blood stem cells.

**Table 1:** Baseline characteristics and lymphocyte subpopulations by the strength of immune response after the three doses of receptor-binding domain (RBD)–tetanus toxoid (TT)-conjugated SARS-CoV-2 vaccine in allogeneic hematopoietic stem cell transplant (allo-HSCT) recipients.

Baseline characteristics			Total	Strength of immune response#		
				Moderate immune response	Strong immune response	*p*-value
**Total, *N***			36	18	18	
Patient**’**s sex, *n* (%)		Female	15 (41.7)	8 (53.3)	7 (46.7)	0.790
		Male	21 (58.3)	10 (47.6)	11 (52.4)	
Donor**’**s sex, *n* (%)		Female	18 (50)	4 (22.2)	14 (77.8)	0.004
		Male	18 (50)	14 (77.8)	4 (22.2)
Primary disease, *n* (%)		AML	27 (75)	13 (48.1)	14 (51.9)	0.791
		ALL	9 (25)	5 (55.6)	4 (44.4)
Patient**’**s age in years (median ± IQR)			42.41 ± 11.4	45.6 ± 11.42	39.16 ± 11.66	0.111
Donor**’**s age in years (median ± IQR)			43.94 ± 11.55	47.61 ± 12.75	40.27 ± 9.15	0.044
Using cyclosporine ≥ 25mg/day at the time of vaccination, *n* (%)			26 (72.2)	12 (46.2)	14 (53.8)	0.463
Using prednisolone ≥ 5mg/day at the time of vaccination, *n* (%)			9 (25)	6 (66.7)	3 (33.3)	0.255
Having GvHD at the time of vaccination¥			15 (41.7)	8 (53.3)	7 (46.7)	0.739
Patient**’**s pre-allo-HSCT PCR-positive COVID-19 status, *n* (%)			15 (41.7)	4 (26.7)	11 (73.3)	0.020
Patient**’**s pre-allo-HSCT SARS-CoV-2 vaccination status, *n* (%)			13 (36.11)	8 (61.5)	5 (38.5)	0.486
Donor**’**s pre-allo-HSCT SARS-CoV-2 vaccination status, *n* (%)			24 (66.7)	11 (45.8)	13 (54.2)	0.360
Patient**’**s ISR pre-allo-HSCT, mean ± SD			1.19 ± 0.73	1.25 ± 0.88	1.13 ± 0.55	0.860
Donor**’**s ISR pre-allo-HSCT, mean ± SD			1.94 ± 0.95	1.49 ± 0.65	2.4 ± 1.00	0.005
Median (IQR) time between allo-HSCT and vaccination in days			133 (107.5 - 228)	130.5 (99 - 202)	133 (115 -231)	0.521
Lymphocyte subpopulations, mean ± SD						
At the first vaccine dose (baseline)	CD4^+^ cells		319.00 ± 208.99	215.79 ± 98.80	399.28 ± 237.87	0.091
	CD8^+^ cells		788.41 ± 461.51	743.62 ± 525.86	823.25 ± 417.13	0.220
	CD4^+^/CD8^+^ ratio		0.46 ± 0.25	0.40 ± 0.25	0.50 ± 0.26	0.280
	CD19^+^ cells		113.77 ± 101.00	74.39 ± 70.02	144.40 ± 122.16	0.090
	CD16^+^ 56^+^ (NK cells)		139.34 ± 91.69	112.70 ± 52.59	160.06 ± 110.34	0.357
At the third vaccine dose	CD4^+^ cells		389.95 ± 193.51	319.45 ± 148.28	448.69 ± 210.58	0.073
	CD8^+^ cells		1082.48 ± 768.64	1207.78 ± 904.20	978.06 ± 642.96	0.708
	CD4^+^/CD8^+^ ratio		0.47 ± 0.25	0.37 ± 0.25	0.55 ± 0.23	0.018
	CD19^+^ cells		180.82 ± 161.94	122.46 ± 125.24	229.45 ± 175.89	0.044
	CD16^+^ 56^+^ (NK cells)		192.95 ± 218.80	147.60 ± 106.81	230.74 ± 278.19	0.929

#Defined based on the median level of ISR after the third vaccine dose.

¥ Including grades 1 or 2 acute GvHD or mild to moderate chronic GvHD.

ISR, immune status ratio; allo-HSCT, allogeneic hematopoietic stem cell transplant; AML, acute myeloid leukemia; ALL, acute lymphoid leukemia; GvHD, graft-versus-host disease; NK, natural killer. The median time between allo-HSCT and the start of vaccination was 133 days (interquartile range 107.5 - 228 days).

Before allo-HSCT, 13 (36.11%) patients and 24 (66.7%) donors had been fully vaccinated against SARS-CoV-2. Information regarding patients’ and donors’ SARS-CoV-2 vaccination history and ISR serologic test results at the time of allo-HSCT is shown in **Table 1**. Information on the use of immunosuppression drugs and the grade and severity of GvHD before the first vaccine dose is also given in **Table 1**. At the time of immunization, 26 patients (72.2%) were receiving calcineurin inhibitors (cyclosporine ≥ 25 mg/day) and nine (25%) were also receiving prednisolone ≥ 5 mg/day but < 0.5 mg/kg/day. Fifteen (41.7%) patients were shown to have grades 1 or 2 acute GvHD, or mild or limited chronic GvHD at the time of vaccination (patients with high-grade acute GvHD or severe chronic GvHD were excluded). The median time between allo-HSCT and the start of vaccination was 133 days (interquartile range 107.5 - 228 days).

### 3.2 Serological outcomes

**Figure 2:**
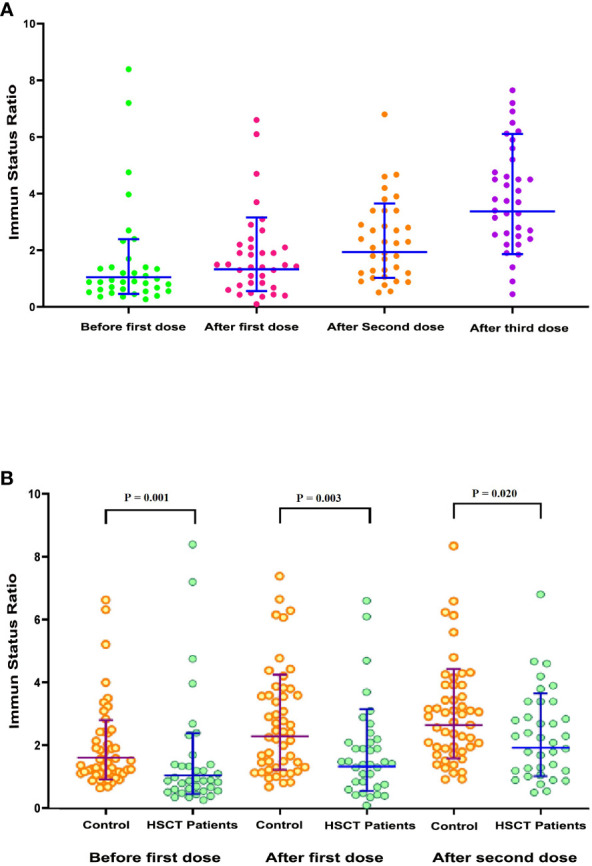
Scatterplot of SARS-CoV-2 immune status ratio (ISR). **(A)** Scatterplot of SARS-CoV-2 ISR in allogeneic hematopoietic stem cell transplant (allo-HSCT) recipients (*n* = 36) during the predefined samples of before the first dose, after the first dose, after the second dose, and after the third dose. **(B)** Comparison scatterplots of SARS-CoV-2 ISR in allo-HSCT patients (*n* = 36) and healthy participants (*n* = 50) during the planned samples before the first dose, after the first dose, and after the second dose.

From January to October 2022, 146 blood samples were obtained from the 36 patients in the study cohort and tested for antibodies against SARS-CoV-2. A scatterplot of the SARS-CoV-2 IgG ISRs during the trial was created (**Figure 2A**).

The mean ISR was 1.55 (95% CI 0.94 to 2.17) at baseline (before the first dose). This markedly increased to 2.32 (95% CI 1.84 to 2.79; *p* = 0.010) and 3.87 (95% CI 3.25 to 4.48; *p* = 0.001) after the second and third doses of vaccine, respectively. Out of 36 patients, 10 (27.17%) had a baseline ISR over the threshold for a positive result in the semiquantitative test. After doses two and three, the proportion of seropositive tests increased to 69.44% and 91.66%, respectively.

As depicted in **Table 2**, taking the pre-vaccination ISRs as the reference group in the multivariable GEE model, the ISR increased dramatically across the second (*p* = 0.041) and third (*p* < 0.001) vaccine doses, regardless of any confounding factors. The donor’s sex (β = –1.33; *p* = 0.010) was the independent factor associated with ISR during the vaccination.

**Table 2:** Univariate and multivariable generalized estimating equation (GEE) model to assess the dynamics of the immune status ratio (ISR) during the three doses of the receptor-binding domain (RBD)–tetanus toxoid (TT)-conjugated SARS-CoV-2 vaccination regimen in allogeneic hematopoietic stem cell transplant (allo-HSCT) recipients, adjusted for confounding factors.

		Univariate		Multivariable	
		β (95% CI)	*p*-value	β (95% CI)	*p*-value
Vaccine doses	Before the first dose	1			
	After the first dose	0.26 (–0.20 to 0.73)	0.260	0.32 (–0.23 to 0.87)	
	After the second dose	0.76 (0.19 to 1.34)	0.010	0.64 (0.02 to 1.26)	0.041
	After the third dose	2.31 (1.62 to 3.00)	< 0.001	2.36 (1.55 to 3.18)	< 0.001
Patient**’**s sex	Female	1			
	Male	1.15 (0.49 to 1.81)	0.001	0.64 (0.01 to 1.28)	0.051
Donor**’**s sex	Female	1			
	Male	–1.68 (–2.52 to –0.83)	< 0.001	–1.33 (–2.33 to –0.32)	0.010
Donor**’**s age (years)		–0.04 (–0.07 to –0.003)	0.040	–0.14 (–0.42 to 0.02)	0.350
Patient**’**s ISR pre-allo-HSCT status		–0.31 (–0.81 to 0.18)	0.210	–0.12 (–0.52 to 0.29)	0.572
Donor**’**s ISR pre-allo-HSCT status		0.37 (–0.05 to 0.78)	0.080	–0.26 (–0.77 to 0.24)	0.313
Patient**’**s pre-allo-HSCT PCR-positive COVID-19 status	No	1			
	Yes	0.88 (0.09 to 1.66)	0.030	0.65 (–2.33 to 1.58)	0.172

ISR, immune status ratio; allo-HSCT, allogeneic hematopoietic stem cell transplant.

Given that the ISR values after the third vaccine dose mostly exceeded the cut-off value for the positive result in the semiquantitative test, it was possible to distinguish between patients with moderate and strong serologic responses based on the median level of the ISR after the third vaccine dose (30). **Table 1** presents the strength of the immune responses (divided into strong or moderate immune response) to the entire course of RBD–TT-conjugated SARS-CoV-2 vaccine following allo-HSCT according to the baseline characteristics of the patients and donors at the time of allo-HSCT and also based on the post-allo-HSCT immune cells’ reconstitution at the time of first and third vaccine doses.

**Figure 3:**
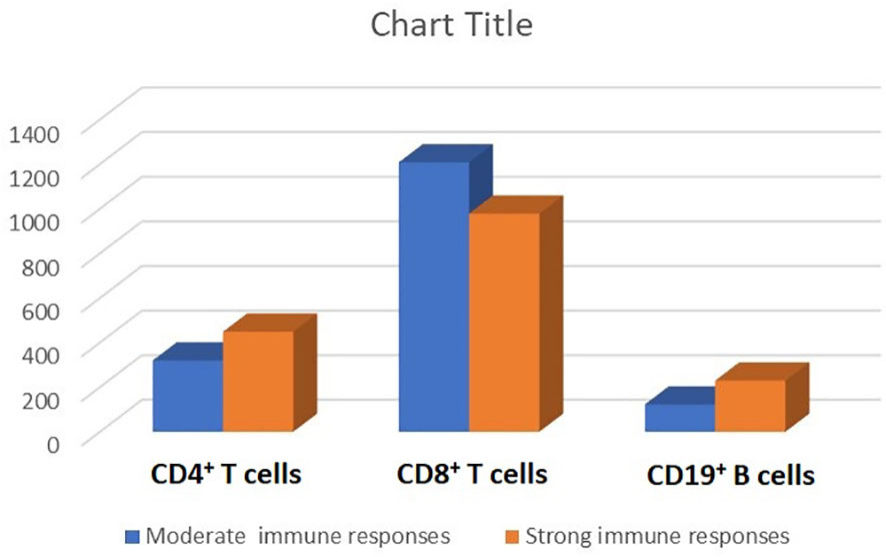
Lymphocyte subpopulations (the mean counts of CD4^+^, CD8^+^, and CD19^+^ cells) stratified by the strength of serologic response (i.e., strong vs. moderate) after three doses of the receptor-binding domain (RBD)–tetanus toxoid (TT)-conjugated SARS-CoV-2 vaccine in allogeneic hematopoietic stem cell transplant (allo-HSCT) recipients.

As depicted in **Table 1**, the strong serologic response following the three doses of the PastoCovac vaccine was more common in recipients who received their allo-HSCT from female donors than those who received their allo-HSCT from male donors (77.8%; *p* = 0.004), and in those with a history of pre-allo-HSCT PCR-positive COVID-19 (73.3%; *p* = 0.020). The median age of donors was lower (i.e., 40.27 vs. 47.61 years; *p* = 0.044) and the mean ISR of donors before allo-HSCT was higher (i.e., 2.4 vs. 1.49; *p *= 0.005) in the strong serologic response group than in the moderate response group. Regarding the correlation of post-allo-HSCT immune cell reconstitution with the strength of the serologic response, as depicted in **Table 1**, the mean counts of CD19^+^ cells and mean CD4^+^/CD8^+^ ratio at the third vaccine dose were significantly higher in patients with a strong serologic response. The mean counts of CD4^+^ cells, CD8^+^ cells, and CD19^+^ cells at the third vaccine dose in the allo-HSCT recipients with strong as compared with moderate serologic responses are shown in **Figure 3**.

Univariate and multivariate logistic regression analyses were performed to determine the predictive indicators of a strong immune response following the third vaccine dose. In the multivariate analysis, the female sex of the donor [odds ratio (OR) 8.67; *p* = 0.028] and a higher donor ISR before allo-HSCT (OR 3.56; *p* = 0.050) remained the two independent predictors of a strong immune response following the third dose of vaccine (**Table 3**).

**Table 3:** Univariate and multivariate logistic regression analysis to determine the predictive factors of a strong serologic response following three doses of the receptor-binding domain (RBD)–tetanus toxoid (TT)-conjugated SARS-CoV-2 vaccine in allogeneic hematopoietic stem cell transplant (allo-HSCT) recipients.

Effect	Univariate		Multivariate	
	Unadjusted OR (95% CI)	P Value	Adjusted OR (95% CI)	P Value
**Patient’s Age**	0.95 (0.89 - 1.01)	0.103	0.94 (0.85 – 1.04)	0.277
**Donor’s Age**	0.94 (0.88 - 1.00)	0.064	0.95 (0.86 – 1.04)	0.273
**Donor’s Sex (Female vs. Male)**	12.25 (2.54 - 58.96)	0.002	8.67 (1.25 -59.9)	0.028
**Donor’s ISR Pre-allo-HSCT**	3.34 (1.38 - 8.07)	0.007	3.56 (0.98 - 12.89)	0.050
**Patient’s Pre-allo-HSCT PCR-positive COVID-19**	5.50 (1.27- 23.69)	0.022	3.09 (0.35 - 27.05)	0.264
**CD4^+^ cells count at vaccination**	1.005 (1.00 -1.01)	0.025	1.006 (0.99 – 1.01)	0.185
**CD19^+^ cells count at vaccination**	1.008 (0.99 -1.01)	0.063	0.99 (0.98 – 1.01)	0.947

OR, odds ratio; CI, confidence interval; ISR, Immune status ratio; allo-HSCT, allogeneic hematopoietic stem cell transplant.

We lacked a parallel control group of healthy participants. However, to compare the serologic response of healthy individuals with this vaccine platform, we used the results of 50 healthy volunteers (22 females and 28 males) with a mean age of 37.92 years (SD 12.62 years) who had received two doses of the RBD–TT-conjugated SARS-CoV-2 vaccine as part of a phase 3 trial at the Pasteur Institute of Iran (IRCT20210303050558N1). For healthy participants, the serologic response was also measured by semiquantitative immunoassay at baseline and 4 weeks (± 1 week) after each vaccine dose. A scatterplot of ISR values at baseline and following the two doses of RBD–TT-conjugated SARS-CoV-2 vaccine for allo-HSCT patients and healthy participants was created (**Figure 2B**). In healthy participants, consistent with allo-HSCT patients, the ISR increased significantly following two vaccination doses (*p* < 0.001). However, the ISR was considerably greater in healthy participants than in allo-HSCT recipients at all three available time points.

### 3.3 Safety

**Figure 4:**
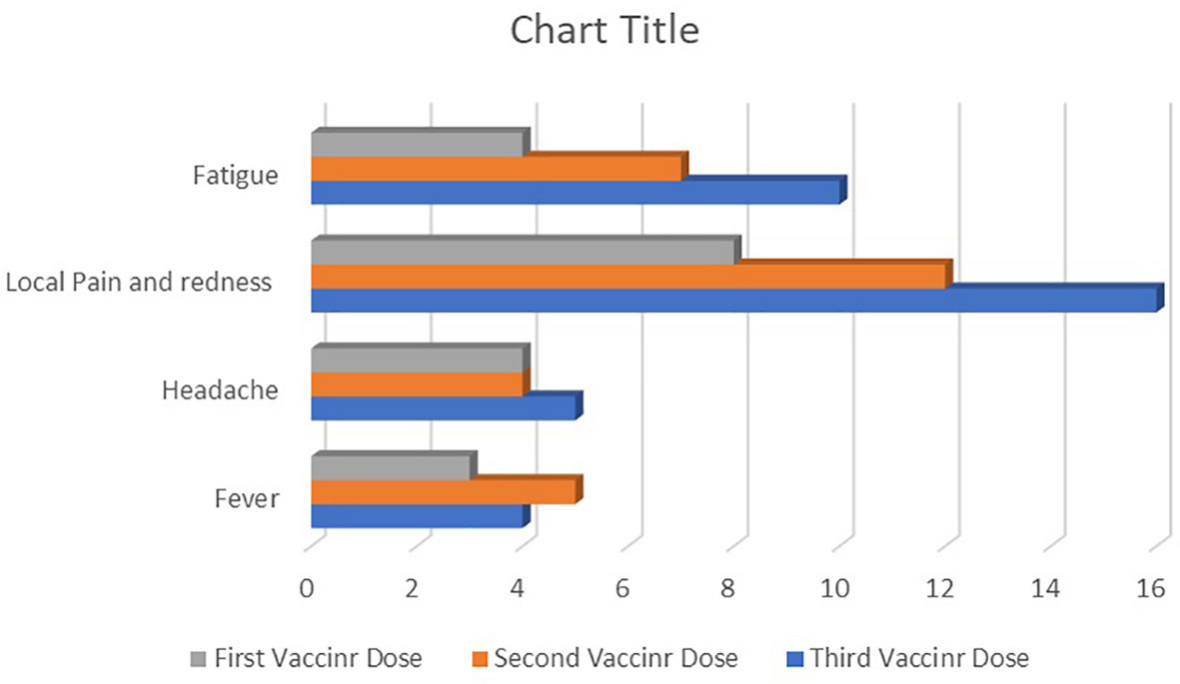
The frequency of reactogenic adverse events after the first, second, and third doses of receptor-binding domain (RBD)–tetanus toxoid (TT)-conjugated SARS-CoV-2 vaccine in allogeneic hematopoietic stem cell transplant (allo-HSCT) recipients.

**Figure 4** provides data about vaccine-related side effects. According to the CTCAE, no participant experienced an AE of grade 3 or 4. After the third dose, AEs occurred more frequently than after the second and first doses. Pain or tenderness at the injection site was the most prevalent AE, occurring in 44.5% of participants after the third dose and 22.2% of participants after the first vaccine dose. Fatigue was the second most frequent AE, seen in 27.7% of participants after the third vaccine dose and 11.1% of participants after the first vaccine dose.

During the study period, over a median follow-up period of 242 days (range 162–309 days) from the beginning of vaccination, one patient died after the first dose because of a relapse of their underlying disease, three patients were excluded after the second dose because of worsening of GvHD, and five patients with documented COVID-19 after the first or second doses were also excluded (**Figure 1**). Over a median follow-up period of 174.5 days (range 106–251 days) from the end of vaccination until the last contact, four PCR-documented COVID-19 infections were reported; these occurred in fully vaccinated patients who presented with mild respiratory symptoms, and no hospitalizations were required.

## 4 Discussion

The impossibility of easy access to the SARS-CoV-2 mRNA- or adenoviral vector-based platforms and the need for timely immunization of allo-HSCT recipients led us to, for the first time, use an accessible and affordable RBD–TT conjugated SARS-CoV-2 vaccine soon after allo-HSCT. Active surveillance showed that the RBD–TT-conjugated vaccine was generally well tolerated in allo-HSCT recipients. Most of the adverse effects were minor and temporary, that is, equivalent to those reported in the general population for this platform (25, 26) and in allo-HSCT recipients who received mRNA-based platforms (7, 8).

Furthermore, as illustrated in **Figure 2A**, the serologic response increased considerably following the three doses of the RBD–TT-conjugated SARS-CoV-2 vaccine in allo-HSCT patients over 3 months post allo-HSCT and without ongoing high-grade GvHD, which is similar to the results reported with mRNA-based platforms (13–15). However, the value of ISR was less than healthy individuals at each available time point (**Figure 2B**). After three doses of the SARS-CoV-2 vaccine, the immune response reached 91.66% at a median duration of 199 days between allo-HSCT and the third vaccine dose. Similarly, Kimura et al. (32) and Watanabe et al. (33) reported that 89.1% and 95% of allo-HSCT patients achieved seroconversion after the third dose of the mRNA-based vaccination, respectively, albeit with a median interval of more than 1 year between allo-HSCT and the third vaccine dose.

Therefore, despite early post-allo-HSCT vaccination, the acceptable seroconversion rate may be partly attributed to the chemical engineering of the vaccine we used. After allo-HSCT, protein-conjugated antigens were found to be more immunogenic than unconjugated ones (34). Notably, TT-conjugated platforms significantly impact early immunogenicity following allo-HSCT (35). The conjugation of RBD to TT has also been shown to promote immune responses to SARS-CoV-2 (23).

Considering the predictors of immune response to the three doses of RBD–TT-conjugated SARS-CoV-2 vaccine early after allo-HSCT, a female donor was a positive predictor for dynamic changes in the ISR during vaccination (**Table 2**) and a strong immune response after the three doses (**Table 3**). A higher level of donor immunity at allo-HSCT was the other predictive factor for a strong immune response after the entire course of post-allo-HSCT vaccines (**Table 3**).

In our study, a stronger immune response after vaccination was not affected by the age of the patients or donors, the length of time between allo-HSCT and vaccination, the presence of GvHD, or the use of immunosuppressive drugs. In contrast, some studies have reported the adverse effects of immunosuppressant medications (33, 36), GvHD (14, 36), and the limited time interval between allo-HSCT and vaccination (15, 33, 36) on the immune response to mRNA-based vaccination in allo-HSCT patients. This disparity may be partly explained by the fact that our patients were vaccinated between 3 and 12 months after allo-HSCT and were reasonably homogeneous in regard to their baseline characteristics. Regarding the optimum time frame for post-allo-HSCT SARS-CoV-2 vaccination, the European and US transplant guidelines advise starting vaccination 3 months after transplantation (5, 6), despite conflicting findings about the impact of the period between allo-HSCT and vaccination on the vaccine-induced immune response (8).

The potential predictive effect of patients’ and donors’ immune conditions at the time of allo-HSCT on antibody production following post-allo-HSCT vaccination was particularly interesting. We found that, as evaluated by ISR shortly before harvesting, donor immunity enhanced the immunological response to SARS-CoV-2 vaccination soon after allo-HSCT. Leclerc et al. proposed that the adoptive transfer of memory cells against SARS-CoV-2 from the vaccinated donors to the recipient induces noticeably higher anti-spike receptor-binding domain [RBD] IgG (IgG (S-RBD)) production after post-allo-HSCT vaccination (37).

Furthermore, our findings revealed that 10 out of 36 allo-HSCT patients (27.78%) exhibited positive ISR before post-allo-HSCT vaccination. Regarding this, Jullien et al. (38) demonstrated the persistence of anti-SARS-CoV-2 antibodies for up to 9 months after allo-HSCT in patients immunized before allo-HSCT. As a result, as the limited published evidence implies, vaccinating donors and recipients against SARS-CoV-2 before allo-HSCT might increase the immune response to prompt post-allo-HSCT SARS-CoV-2 revaccination (37–40).

Similar to the results of mRNA-based vaccines in allo-HSCT patients (33, 36, 41, 42), we found that CD4^+^ T-cell count and CD4^+^/CD8^+^ ratio after the first and third vaccines were correlated with a serologic response after the third dose. The CD4^+^/CD8^+^ ratio and CD19^+^ B-cell counts were also associated with the strength of immunological response following the third dose (**Table 1**). Similarly, Clémenceau et al. (43) and Ram et al. (44) suggest that innate immune response is crucial to the immunogenicity of the COVID-19 vaccine, especially in patients vaccinated in the first year following transplantation.

Our findings revealed a positive prognostic effect of donor sex on the immunological response to SARS-CoV-2 vaccination following allo-HSCT. Recent studies in the general population (45, 46) or allo-HSCT patients (9) have demonstrated an association between the female sex and a higher immune response to the SARS-CoV-2 vaccine, which may be reflected in the more robust T-cell activation in females than in males during COVID-19 infection or vaccination (46). To our knowledge, the sex-related SARS-CoV-2 immunity in allo-HSCT patients has only been examined for the recipient’s sex. However, the post-allo-HSCT immune response largely depends on the donor’s origin.

The following observations should be made on the limits and merits of the study. Our investigation faced constraints owing to its small sample size and single-center design. We could not accurately measure the concentration of anti-S antibodies using the semiquantitative method. We could not form a healthy control group because most of the healthy population had already received the SARS-CoV-2 vaccine. We could not assess cellular immunity as were unable to access the functional assay kit for SARS-CoV-2-specific T-cell responses.

Regarding the study’s strengths, our study was conducted on a homogeneous cohort of adult acute leukemia patients who underwent allo-HSCT with the same myeloablative conditioning regimen and graft source. In a constrained window of between 3 and 12 months after transplantation, all patients received three doses of an innovative and widely available RBD–TT-conjugated SARS-CoV-2 vaccine at 4-week intervals. All patients were monitored for reactogenic and non-reactogenic adverse effects regularly and actively.

### 4.1 Conclusion

In conclusion, we found that three doses of the novel RBD–TT-conjugated SARS-CoV-2 vaccine are safe, highly immunogenic, and affordable for allo-HSCT patients. Our findings indicate that allo-HSCT recipients, particularly in endemic regions, should be offered a full course of COVID-19 vaccination starting 3 months after allo-HSCT, assuming that they do not have high-grade acute GVHD. This suggestion is in line with European and US transplant guidelines. However, following the immunization of allo-HSCT patients, active surveillance is necessary. We further believe that pre-allo-HSCT SARS-CoV-2 immunization in donors may enhance subsequent post-allo-HSCT seroconversion in patients who receive the entire course of the SARS-CoV-2 vaccination during the first year post-allo-HSCT.

## Data availability statement

The raw data supporting the conclusions of this article will be made available by the authors, without undue reservation.

## Ethics statement

The studies involving human participants were reviewed and approved by The Ethics Committee of Tehran University’s Hematology, Oncology and Stem Cell Transplantation Research Center (IR.TUMS.HORCSCT.REC.1400.021). The patients/participants provided their written informed consent to participate in this study.

## Author contributions

MV and MaB contributed to the conception and design of the study. LSA organized the database. FA performed the statistical analysis. MaB wrote the first and final draft of the manuscript. BC, MA, and MRS performed the laboratory evaluation. FA carried out the review and edited the manuscript. TB and MaB contributed to clinical management. AB contributed to the vaccine platform design. All authors contributed to the article and approved the submitted version.
